# Gegen Qinlian Decoction Relieves Ulcerative Colitis via Adjusting Dysregulated Nrf2/ARE Signaling

**DOI:** 10.1155/2022/2934552

**Published:** 2022-04-25

**Authors:** Chuan Lin, Zehua Zhou, Lijun Zhang, Hongqing Wang, Jie Lu, Xinhong Wang, Rui An

**Affiliations:** Shanghai University of Traditional Chinese Medicine, Shanghai 201203, China

## Abstract

**Objective:**

Oxidative stress has been proven to be essential in the pathogenesis of ulcerative colitis (UC). Therefore, this study was designed to investigate the effect of Gegen Qinlian decoction (GQ) on the Nrf2 pathway in the treatment of UC and explore the potential mechanism.

**Methods:**

The UC rat model was induced by 5% dextran sodium sulfate (DSS) aqueous solution, and UC rats were treated with GQ orally. The effect of GQ on UC rats was recorded. Human clonal colon adenocarcinoma cells (Caco-2) stimulated by tumor necrosis factor-*α* (TNF-*α*) were employed in this study. After being stimulated with TNF-*α* for 2 hours, Caco-2 cells were cultured with GQ or its major components (puerarin, baicalin, berberine, and liquiritin) for 22 hours. In addition, the Nrf2 gene of Caco-2 cells was silenced and then cultured with GQ for 22 hours. The contents of superoxide dismutase (SOD), glutathione peroxidase (GSH-PX), and malondialdehyde (MDA) in colonic tissues and Caco-2 cells were detected by assay kits. Reactive oxygen species (ROS) in Caco-2 cells were analyzed by flow cytometry. Quantitative real-time PCR and western blot were employed to detect the mRNA and protein expression of Nrf2 and its related target genes in colon tissues and Caco-2 cells.

**Results:**

GQ alleviated the injured colonic mucosa and activated the expression of Nrf2 in UC rats. In TNF-*α* stimulated Caco-2 cells and Nrf2 silenced Caco-2 cells, GQ also reversed the inhibitory effect of Nrf2. Furthermore, the major components of GQ could activate Nrf2 signaling in TNF-*α* stimulated cells as well. Moreover, the contents of SOD, GSH, MDA, and ROS were restored to normal after treatment with GQ or its major components. Among these components, puerarin, berberine, and liquiritin appear to have a better effect on activating Nrf2 in *vitro.* Overall, GQ can alleviate UC by increasing the activity of Nrf2/ARE signaling and enhancing the effect of antioxidant stress.

## 1. Introduction

Currently, there is a high incidence rate of gastrointestinal diseases. The prevalence of ulcerative colitis (UC) in China is significantly increasing year by year [1], and the patients are distributed in all age groups. UC is a chronic nonspecific inflammatory disease of the colon and rectum whose etiology remains unknown. Normally, the lesions of the colon are limited to the mucosa and submucosa of the large intestine, most of which are located in the sigmoid colon and rectum. Worse, they can even extend to the descending colon or the whole colon. The progression of UC is usually long and easily recurrent. In addition, colorectal cancer may occur after long-term chronic intestinal inflammation. Although environmental factors, genetic background, and immunological dysfunction have been reported to be the main causes of UC, the precise mechanisms involved in the initiation and development of UC have not been completely elucidated yet. Considering many chemical medications for UC such as aminosalicylic acid, immunosuppressive agents, and biological agents have apparent limitations, including a substantial economic burden and undesirable side effects, some natural medicines with fewer side effects are worthy of being taken into consideration and further investigation.

Gegen Qinlian decoction (GQ), a traditional Chinese medicine prescription, consists of four herbs which are *Scutellaria* Radix, *Coptis* Rhizoma, *Pueraria lobata* Radix, and Glycyrrihiza Radix et Rhizoma. Nowadays, it is commonly used in the treatment of acute enteritis, bacillary dysentery, enteric typhoid, and gastrointestinal cold. Compared to other chemical drugs, GQ has exhibited apparent therapeutic effects and only has a few side effects on UC. GQ can exert multiple therapeutic effects against UC via toll-like receptor 4 (TLR4)/nuclear transcription factor-*κ*B (NF-*κ*B) signaling pathway [2], interleukin-6 (IL-6)/Janus tyrosine kinase 2 (JAK2)/signal transducer, and activator of transcription 3 (STAT3) signaling pathway [3], and Notch signaling pathway [4]. Moreover, GQ can regulate intercellular molecular transmission and alter the immune response as well as oxidative stress to protect the intestinal mucosal barrier.

NRF2 (nuclear factor (erythroid-derived 2)-like 2) is an essential transcriptional factor [5] of countering oxidative stress and is the central regulator of maintaining redox homeostasis in cells. Nrf2 can not only alleviate the injury of cells induced by reactive oxygen species (ROS) but also maintain stability in cells by antioxidation [6]. UC is deemed to be caused by oxidative stress, and the recovery mechanism of UC is related to the expression of Nrf2 [7–11].

Thus, this study was formulated to reveal the therapeutic mechanism by which GQ regulates inflammatory cytokines and oxidative stress via promoting the Nrf2/ARE signaling pathway. This study better illustrated the antioxidant stress effect of GQ and confirmed the relationship between GQ treatment of UC and the Nrf2 signaling pathway.

## 2. Materials and Methods

### 2.1. Preparation of GQ

All the raw herbal medicines of GQ were obtained from Shanghai Kangqiao traditional Chinese medicine decoction pieces Co. Ltd. (Shanghai, China) and were identified by the following per under the Pharmacopoeia of the People's Republic of China (2020 edition). The GQ was prepared according to the previous report [2] and concentrated to 1 g/ml for experiments in *vivo*. For experiments in *vitro,* the GQ solution was dissolved in dimethyl sulfoxide (DMSO) and concentrated to 125 *μ*g/ml.

### 2.2. Experimental Animals

Male Sprague–Dawley rats (180 ± 20 g) were obtained from Shanghai Laboratory Animal Co. Ltd. (SLAC) (production license: SCXK Shanghai 2017-0005). Rats were raised in the Experimental Animal Center of Shanghai University of Traditional Chinese Medicine, adequately supplied of standard rat chow and water, maintained at 25°C, 12 h : 12 h light-dark cycle. The study protocol was approved by the Experimental Animal Center of the Shanghai University of Traditional Chinese Medicine.

### 2.3. Experimental Induction of UC Model and Treatment Schedule

UC rats were induced with 5% (w/v) DSS [12] dissolved in drinking water continuously for 7 days, while the control (CON) group rats drank water without DSS (*n* = 8 rats in each group). After the UC model was successfully established, we divided the rats into four groups: the CON group, the DSS group, the GQ group, and the sulfasalazine (SASP) [13] group. GQ (17 g/kg) and SASP (350 mg/kg) were administered by gavage with physiological saline as the vehicle from day 8 to day 14 (the doses were estimated according to the clinical dose for humans). The CON and DSS groups received the same amount of physiological saline. Body weight, stool consistency, the presence of gross blood in feces, and the anus were measured generally every day. The disease activity index (DAI) was calculated as previously described [14] on day 15. On day 15, the rats were euthanized and the entire colons were quickly removed.

### 2.4. Preparation of Colonic Tissue Samples

After excising the colonic tissue, the length was measured. Then it was rinsed with ice-cold physiological saline and the harvested tissue was fixed in 4% paraformaldehyde for histopathology. The remaining colonic tissue was stored in the laboratory at −80°C for further experiments.

### 2.5. Histological Studies

Colonic segments were fixed in 10% normal buffered formalin [15], sectioned at 4 *μ*m thickness with a paraffin microtome, and mounted on microscope slides. Sections were stained with HE (hematoxylin and eosin) and a Colon Histopathological Score (HPS) [16] was calculated to evaluate the damages of epithelium mucosa and inflammatory infiltration.

### 2.6. Cell Culture and Sample Treatment

The human clonal colon adenocarcinoma cells (Caco-2) were purchased from iCell Bioscience Inc. (Shanghai, China) and maintained in minimum essential medium (MEM) supplemented with 20% heat-inactivated fetal bovine serum (FBS) (Gibco, USA), 100 U/ml penicillin and 100 *μ*g/ml streptomycin at 37°C under a humidified atmosphere of 5% CO_2_. Based on the previous literature [4, 17], cells in the model group (MOD) were stimulated with TNF-*α* at the concentration of 80 *μ*g/L. The cytotoxicity experiment verified that the concentration of GQ was 18.75 *μ*g/ml and did not affect Caco-2 cell activity (Figure S1). The model and therapeutic groups were stimulated with TNF-*α* (80 *μ*g/L) for 2 h. Then, the therapeutic groups were treated with GQ (L-GQ: 6.25 *μ*g/ml, M-GQ: 12.5 *μ*g/ml, and H-GQ: 18.75 *μ*g/ml) for 22 h, and berberine (20 *μ*M) was used as a positive control [2].

To assess the regulation of major components [4] in GQ on Nrf2 signaling in Caco-2 cells, cells were seeded in 6-well plates and divided into control (CON), MOD, puerarin (PUE), baicalin (BA), berberine (BBR), liquiritin (LQ), and GQ groups. The concentrations of these monomers used in *vitro* were selected according to the previous report [4]. The model and therapeutic groups were stimulated with TNF-*α* (80 *μ*g/L) for 2 h. Then, the therapeutic groups were treated with PUE (20 *μ*M), BA (20 *μ*M), BBR (20 *μ*M), LQ (20 *μ*M), and GQ (18.75 *μ*g/ml) for 22 h.

### 2.7. Effect of GQ on Nrf2 Gene Silenced Caco-2 Cells

The Nrf2 gene was silenced by transfection of siRNA fragments [18, 19]. After 24–48 h, 100*x* fluorescence photos were taken with a microscope and the transfection result was detected by qRT-PCR. Then, the transferred cells were cultured with GQ (18.75 *μ*g/ml) for 22 h.

### 2.8. Assay of SOD, MDA, and GSH Levels

The total contents of SOD, MDA, and GSH in the colon homogenate and Caco-2 cells were examined by colorimetric analysis following the instruction. The protein content was assayed by the BCA Protein Quantification Kit of Yisheng Biotech Co., Ltd. (Shanghai, China), and then the results were expressed as activity units per mg.

### 2.9. Measurement of Intracellular ROS

Cells treated in 2.6 and 2.7 were collected. After being washed twice with PBS, the cells were incubated with 20 *μ*M DCFH-DA and 10 *μ*g/ml Hoechst 33258 for 30 min, and then the measurement of intracellular ROS was analyzed by flow cytometry immediately.

### 2.10. RNA Isolation and qRT-PCR Assay

Total RNA of colonic tissues and Caco-2 cells was isolated *via* Trizol. The concentration of total RNA was determined by a nucleic acid protein detector (Tables S1–S4). Reverse transcription with PCR (qRT-PCR) was conducted [20]. The primer sequences used for qRT-PCR are listed in Table 1. The gene expression levels of Keap1, Nrf2, NQO1, and HO-1 were calculated by the 2^−∆∆Ct^ method. Expression levels of the mRNAs of interest were normalized to those of their endogenous reference gene *β*-actin.

### 2.11. Western Blot

The total protein was extracted from colonic tissues and Caco-2 cells by using RIPA buffer with protease inhibitors. The protein expression [21] of Nrf2 (110 kDa), Keap1 (69 kDa), NQO1 (32 kDa), HO-1 (31 kDa), GCLC (73 kDa), GCLM (31 kDa), GAPDH (36 kDa), and *β*-actin (42 kDa) were detected *via* the AI 600 ultrasensitive multifunctional imager (GE, USA) and were quantified by ImageJ software. The relative expression of the target bands was normalized by glyceraldehyde-3-phosphate dehydrogenase (GAPDH) or *β*-actin.

### 2.12. Statistical Analysis

Statistical analysis in each study (at least three repeated experiments) was determined by one-way ANOVA with Prism 9.0 software. All data were presented with mean values and SE and values of *P* < 0.05 were considered to be statistically significant.

## 3. Results

### 3.1. GQ Alleviated UC Rats via Upregulation of Nrf2 Expression

**Figure 1:**
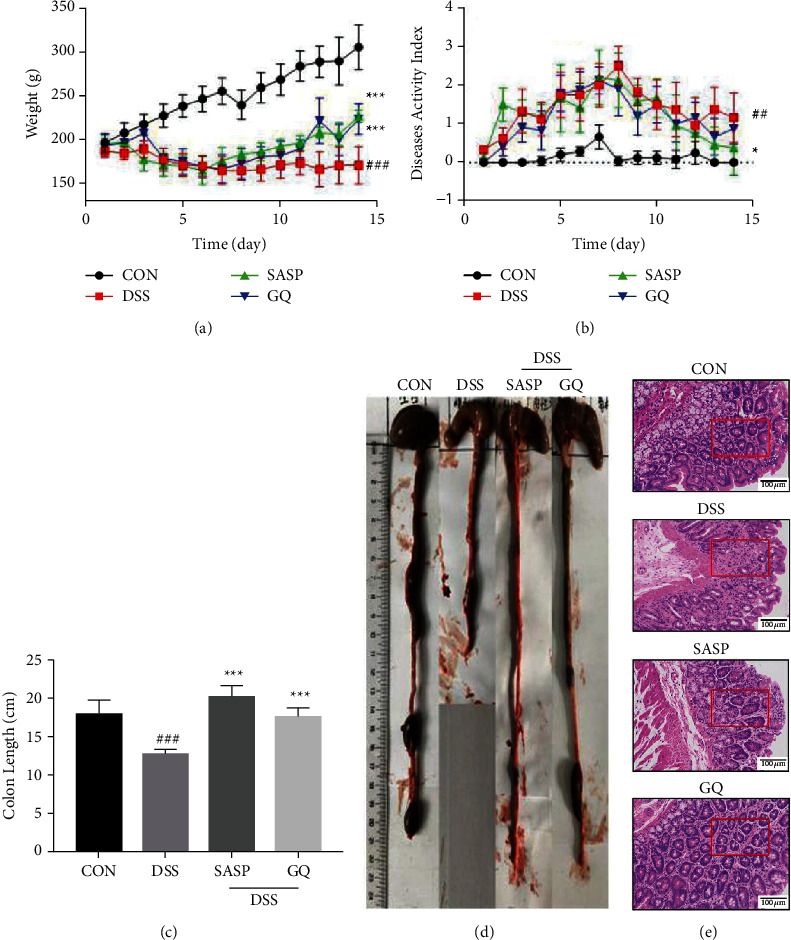
GQ alleviated UC induced by DSS (*n* = 8 in each group). (a) Body weight of every rat was measured per day. (b) DAI of four groups. (c, d) The colon length of every rat. (e) The colon was stained with HE (200*x*). ^#^*P* < 0.05, ^##^*P* < 0.01, and ^###^*P* < 0.0001 versus control group. ^*∗*^*P* < 0.05, ^*∗∗*^*P* < 0.01, and ^*∗∗∗*^*P* < 0.0001 versus model group.

UC Rats induced by DSS were used to evaluate the effect of treatment of GQ. Except for the CON group, the weight loss of each model rat was the same (Figure 1(a)). After oral administration of SASP and GQ, the weight loss of rats in the SASP group and GQ group was alleviated. From day 8, the body weight in the SASP and GQ groups increased dramatically and recovered. On the 14th day, the average weight of rats in the CON group was 304.79 ± 25.24 g, which was 1.77-fold higher than that in the DSS group. In addition, the weight of rats in the SASP group and GQ group was 1.32-fold and 1.30-fold that of the DSS group, respectively. The DAI score was lower in the SASP and GQ groups than in the DSS group (Figure 1(b)). The colon length of rats in the DSS group was significantly shorter than in other groups (Figures 1(c) and 1(d)).

Moreover, according to the colon tissue section and HPS (Figure 1(e), Table 2), in the CON group, the colonic mucosal epithelium, glands, and goblet cells were complete, the structure was clear, the recess was not damaged, the epithelial cells were arranged orderly, and there was no inflammatory cell infiltration. In the DSS group, most of the crypts, glands, and epithelial structures were incomplete, intestinal mucosa necrosis and abscission, and inflammatory cell infiltration. After drug intervention, compared with the DSS group, the intestinal glands became longer and richer, recovered to a certain extent, and the injury degree of the crypt structure in the mucosal layer was alleviated. In line with the previous report [2–4], UC rats can be relieved by GQ.

Subsequently, the effect of GQ on SOD, GSH, and MDA of the colon tissue was assessed. As shown in Table 2, treatment with GQ and SASP could significantly promote SOD and GSH activity in the colon tissues. Compared to the DSS group, the content of MDA in the treatment groups dramatically decreased.

**Figure 2:**
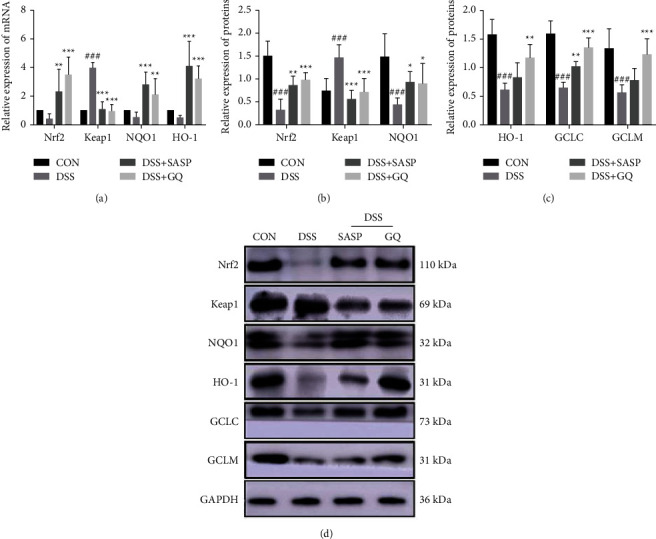
GQ promoted Nrf2 expression in vivo (*n* = 8 in each group). The mRNA levels (a) of Nrf2, Keap1, NQO1, and HO-1 from colon mucosa were detected by the RT-qPCR analysis. The relative protein expression (d) of Nrf2 (b), Keap1 (b), NQO1 (b), HO-1 (c), GCLC (c), and GCLM (c) from the colon tissue of each rat in different groups. GAPDH served as internal control. ^#^*P* < 0.05, ^##^*P* < 0.01, and ^###^*P* < 0.0001 versus CON group. ^*∗*^*P* < 0.05, ^*∗∗*^*P* < 0.01, and ^*∗∗∗*^*P* < 0.0001 versus DSS group.

Furthermore, qRT-PCR analysis (Figure 2(a)) showed that the mRNA expression of INrf2 (Keap1) was upregulated markedly in the DSS group but was suppressed in the GQ group, while the mRNA expressions of Nrf2, NQO1, and HO-1 were decreased in the DSS group and increased in the GQ and SASP groups. The results of western blot analysis (Figures 2(b)–2(d)) were consistent with those of qRT-PCR. The protein expression of GCLC and GCLM decreased in the DSS group and increased in the GQ and SASP groups, which was by following per under the changes in Nrf2 expression. These results collectively showed that GQ promoted the upregulated Nrf2 signaling in UC rats and promoted antioxidant stress in colon tissue to repair the colonic mucosa.

### 3.2. GQ Promoted the Expression of Nrf2 Signaling In Vitro

To confirm that GQ can promote the expression of the Nrf2 signal, Caco-2 cells were stimulated with TNF-*α* for 2 hours and cultured with different concentrations of GQ or BBR for 22 hours. As shown in Table 3, the content of SOD and GSH in the MOD group was reduced dramatically compared to the CON group. However, the levels of SOD and GSH notably increased in groups treated with GQ and BBR. On the contrary, the levels of MDA were reduced remarkably in the GQ and BBR groups compared to the MOD group. The production of ROS was also increased significantly with the stimulation of TNF-*α* in Caco-2 cells, while it was reduced by GQ and BBR treatment.

**Figure 3:**
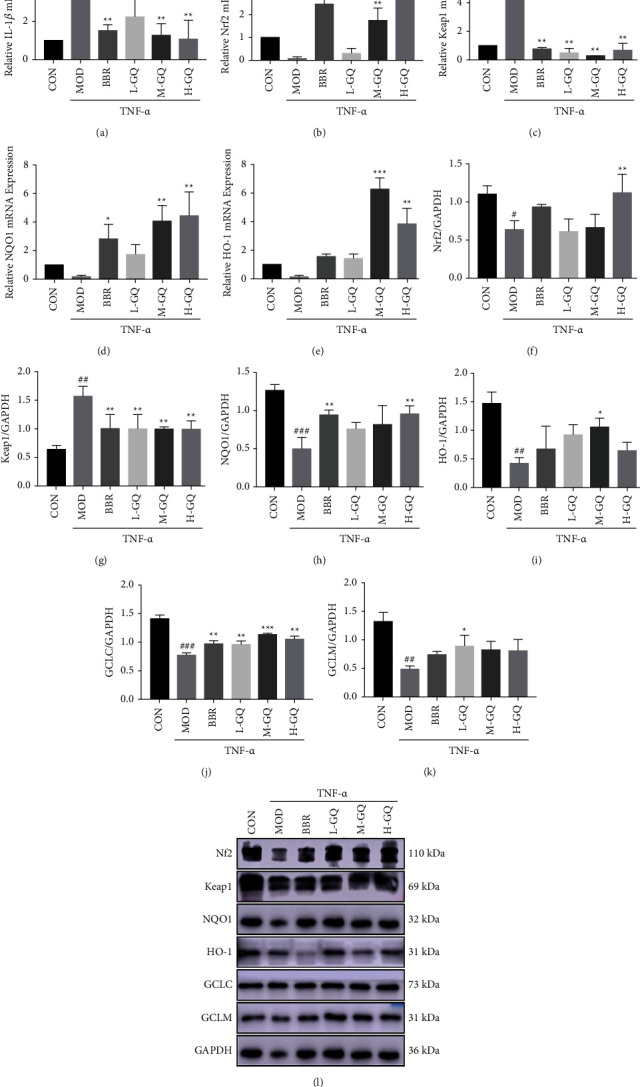
Antioxidant stress effect of GQ in vitro. GQ was anti-inflammatory (a) and promoted the mRNA (b–e) and protein (f–l) expression of the Nrf2 signaling pathway in vitro. ^#^*P* < 0.05, ^##^*P* < 0.01, and ^###^*P* < 0.0001 versus CON group. ^*∗*^*P* < 0.05, ^*∗∗*^*P* < 0.01, and ^*∗∗∗*^*P* < 0.0001 versus MOD group.

As shown in Figure 3, without treatment of GQ or BBR, TNF-*α* increased the level of IL-1*β* (Figure 3(a)) and Keap1 (Figure 3(c)) by almost 4.65- and 4.37-fold compared to the CON group. In addition, the mRNA expression of Nrf2, NQO1, and HO-1 (Figures 3(b), 3(d), and 3(e)) was reduced with TNF-*α* induction. And GQ and BBR treatments increased the mRNA expression of those genes, respectively, compared to the MOD group. The results of western blotting (Figures 3(f)–3(l)) were consistent with the qRT-PCR results, indicating that GQ upregulated Nrf2 signaling in conventional inflammation in *vitro*. GQ with a concentration of 18.75 *μ*g/L was utilized for follow-up experiments.

### 3.3. Effect of GQ on the Silencing Caco-2 Cells of the Nrf2 Gene

To further confirm the effect of GQ on Nrf2 signaling, a transfection vector with a siRNA fragment was used. The silencing of Nrf2 in Caco-2 cells was verified by qRT-PCR analysis. The result showed that (Table 4), in the case of Nrf2 being silenced, the rising content of SOD, GSH, MDA, and ROS secretion were reversed, compared to untreated groups.

**Figure 4:**
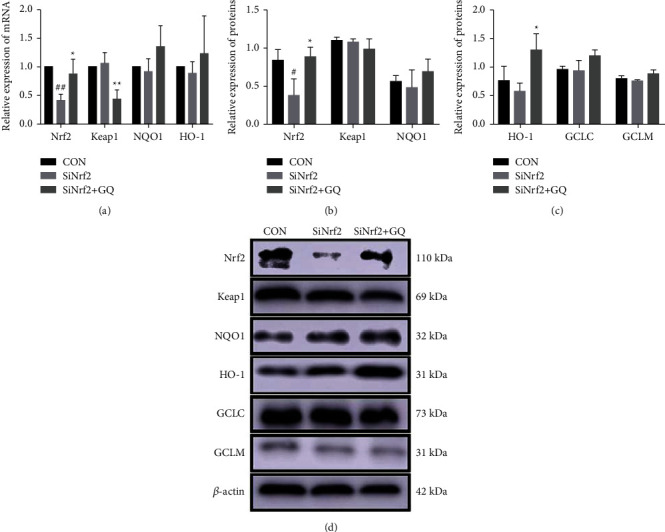
GQ activated Nrf2 gene expression in Nrf2 gene silenced cells. GQ activated the mRNA (a) and protein (b–d) expression of the Nrf2 signaling pathway in it. ^#^*P* < 0.05, ^##^*P* < 0.01, and ^###^*P* < 0.0001 versus CON group. ^*∗*^*P* < 0.05, ^*∗∗*^*P* < 0.01, and ^*∗∗∗*^*P* < 0.0001 versus SiNrf2 group.

Moreover, after the Nrf2 gene was silenced, qRT-PCR analysis (Figure 4(a)) showed that Nrf2 gene silencing had little effect on the gene expression of keap1, NQO1, and HO-1. Nrf2 mRNA expression was significantly activated after GQ treatment. In addition, GQ significantly decreased Keap1 mRNA expression compared with the untreated group. The expression of Nrf2 protein (Figures 4(b) and 4(d)) was consistent with the results of qRT-PCR. GQ significantly promoted the expression of HO-1 protein (Figures 4(c) and 4(d)).

### 3.4. The Effect of the Major Components in GQ on Caco-2 Cells Stimulated with TNF-*α*

As illustrated in Table 5, BBR upregulated the level of SOD in Caco-2 cells stimulated with TNF-*α*, PUE, and BBR upregulated the level of GSH in them. On the other hand, PUE, BA, BBR, and LQ, to different degrees, downregulated the levels of MDA and ROS.

**Figure 5:**
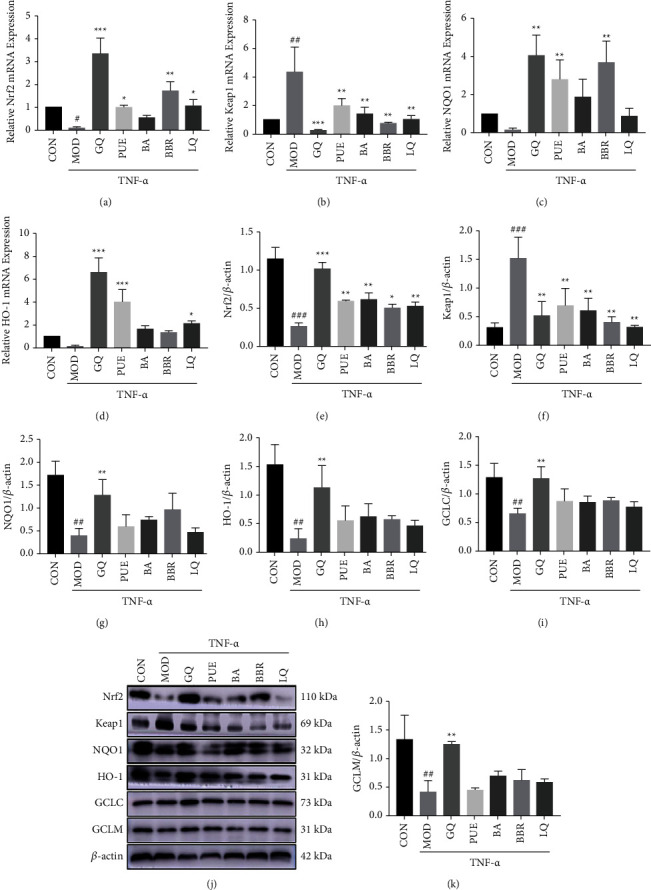
Major components of GQ promoted the mRNA (a–d) and protein (e–k) expressions of the Nrf2 signaling pathway in cells. ^#^*P* < 0.05, ^##^*P* < 0.01, and ^###^*P* < 0.0001 versus CON group. ^*∗*^*P* < 0.05, ^*∗∗*^*P* < 0.01, and ^*∗∗∗*^*P* < 0.0001 versus MOD group.

Moreover, PUE, BA, BBR, and LQ decreased the mRNA expression of Keap1 (Figure 5(b)) induced by TNF-*α*, while PUE, BBR, and LQ increased the mRNA expression of Nrf2 (Figure 5(a)). In addition, PUE and BBR could significantly upregulate the mRNA expression of NQO1 (Figure 5(c)). Besides, PUE and LQ could promote HO-1.

(Figure 5(d)) mRNA expression and western blot analysis (Figures 5(e)–5(k)) showed that those components could significantly upregulate the protein expression of Nrf2, but downregulate the protein expression of Keap1. BBR could significantly upregulate the expression of the NQO1 protein. Though other components could upregulate the expression of HO-1, GCLC, and GCLM, there was no significant difference compared to the MOD group.

## 4. Discussion

In previous clinical studies, GQ had a good alleviation effect on UC [4], indicating that traditional Chinese medicine is a potential strategy that could be used as a complementary therapy for UC. Therefore, further experimental research is necessary to supplement the mechanism of GQ in the treatment of UC. Our studies indicate that the therapeutic effect of GQ on UC is related to antioxidant stress [2, 22]. However, the molecular mechanism of GQ in restoring the damaged intestinal mucosa remains *via* antioxidant stress remains unclear. One of the major cellular defenses against oxidative stress or electrophile stimulation is activating Keap1-Nrf2-ARE signaling [23, 24], which could eliminate ROS and electrophiles by upregulating antioxidant proteins.

In this study, the in *vivo* evidence confirmed that GQ can alleviate UC rats by upregulating Nrf2 signaling. Briefly, in UC rats, GQ treatment decreased Keap1 protein and increased Nrf2 protein. This is related to the conformational change of Keap1 after drug treatment, which separates Nrf2 from Keap1 [25]. Then, the activated Nrf2 entered into the nucleus and combined with the antioxidant response element (ARE) to initiate the expression of downstream signals such as phase II detoxification enzymes (NQO1), antioxidant proteins (HO-1), GCLC, GCLM, ubiquitinates, and proteasomes. NQO1 plays a vital role in antioxidant stress by maintaining the reduction state of coenzyme Q and vitamin E [26]. HO-1 has a protective effect on the body tissues and maintains the redox homeostasis [27–29] in the body. GCLC and GCLM formed glutamyl-L-cysteine ligase, which catalyzed the de novo synthesis of GSH [30]. GSH [31, 32] is very important for maintaining the structural and functional integrity of the intestine, inhibiting the formation of free radicals, or eliminating the formed free radicals. SOD, another downstream enzyme of the Nrf2 pathway, which exists in mitochondria and cytoplasm, maintains the redox balance of tissues and removes the superoxide anion produced in the body. MDA is the source of the elevated oxidative stress injury in the colon tissue of DSS-induced colitis rats. In this experiment, GQ activated Nrf2 as well as its downstream antioxidant proteins and enzymes in the colon of UC rats. Furthermore, GQ significantly reduced the level of MDA. It revealed for the first time that GQ treatment could activate the Nrf2 signaling pathway in the colon of DSS-induced UC rats.

In UC rats, once the mucosal barrier is destroyed by DSS, the submucosa will be infected by gastrointestinal bacteria, leading to a great secretion of ROS [33, 34]. Once Nrf2 signaling is triggered, it will accompany the production of proinflammatory mediators. Herein, we established both UC rats model induced by DSS and Caco-2 cell stimulated by TNF-*α* to dissect the molecular mechanism of GQ. In line with the results of the in *vivo* experiment, in the study of activating the Nrf2 signaling in *vitro*, relevant target genes and target proteins are activated as well. In addition, the contents of ROS and MDA are reduced.

Based on the results of both in *vivo* and in *vitro*, the anti-inflammatory effect of GQ seems to be related to the antioxidant stress induced by activated Nrf2. To further confirm that the effect of GQ on alleviating inflammation was via regulating the Nrf2 pathway, siRNA was used to silence the Nrf2 gene in Caco-2 cells and then treated with GQ. Experimental results show that GQ can promote the expression of inhibited Nrf2 genes and proteins in Caco-2 cells and inhibit Keap1 gene expression. Based on this, GQ could change the expression of the Keap1 gene and its protein conformation, thereby activating the Nrf2 signaling pathway.

In our previous experiments, the composition and contents of GQ and its single herbal medicine were determined (Table S5). And it also verified that the lack of a single herbal medicine in GQ will weaken its curative effect. Even when the dose of a single drug was increased, the effect was not as good as the combined effect of compound drugs. GQ is composed of several components, such as puerarin, baicalin, liquiritin, daidzein, and berberine, and each major compound may have a specific function. To explore which component of GQ plays a dominant role in regulating the Nrf2 pathway, Caco-2 cells stimulated with TNF-*α* were treated with puerarin, baicalin, berberine, and liquiritin, respectively. These major components of GQ have similar promoting effects on the expression of Nrf2 protein; however, the therapeutic effect of these monomers is not as good as GQ. In addition, these monomer components are inferior to GQ in promoting the expression of proteins downstream of Nrf2 signaling. Based on our results, it can be said that these main components in GQ can promote the Nrf2 signal and alleviate DSS-induced UC through a synergistic effect.

## 5. Conclusions

In conclusion, this research revealed for the first time that GQ restored the regeneration and homeostasis of the colonic mucosa *via* enhancing Nrf2 signaling in UC models. Meanwhile, GQ had an anti-inflammatory effect in *vitro* in the same way. In addition, it proved that GQ had a greater pharmacological effect than puerarin, baicalin, liquiritin, daidzein, and berberine which are the major components in GQ to regulate the Nrf2 pathway. This study may provide alternative therapeutic approaches to UC by restoring the homeostasis of colonic mucosa.

## Figures and Tables

**Table 1:** Primer sequences used for real-time quantitative PCR (sequences 5′–3′).

Gene	Species	Forward	Reverse
Nrf2	Rat	TGACAATGAGGTTTCTTCGGCTACG	GGAGAGGATGCTGCTGAAGGAATC
	Homo	TGACAATGAGGTTTCTTCGGCTACG	GGAGAGGATGCTGCTGAAGGAATC

Keap1	Rat	GCGGTGAGAAGAGCCCTGATTG	GCCTTCCTTATACGCCAGAGATGAC
	Homo	GAGAGGAACGAGTGGCGAATGATC	AACAGGTACAGTTCTGCTGGTCAATC

NQO1	Rat	GCGGTGAGAAGAGCCCTGATTG	GCCTTCCTTATACGCCAGAGATGAC
	Homo	CGCAGACCTTGTGATATTCCAG	CGTTTCTTCCATCCTTCCAGG

HO-1	Rat	GGAACTTTCAGAAGGGTCAGGTGTC	GCTGTGTGGCTGGTGTGTAAGG
	Homo	CAGCATGCCCCAGGATTTG	AGCTGGATGTTGAGCAGGA

IL-1*β*	Homo	CCAGGGACAGGATATGGAGCA	TTCAACACGCAGGACAGGTACAG

*β*-actin	Rat	CTGTGTTGTCCCTGTATGCCTCTG	GGAACCGCTCATTGCCGATAGTG
	Homo	CCTGACTGACTACCTCATGAAG	GACGTAGCACAGCTTCTCCTTA

**Table 2:** Effect of GQ on HPS, SOD, GSH, and MDA in the colon tissue ($\bar{x}\pm s$, *n* = 8).

Groups	HPS	T-SOD/U·mL^−1^	GSH/*μ*mol·L^−1^	MDA/*μ*mol·L^−1^
CON	3.60 ± 0.37	386.80 ± 8.72	218.90 ± 31.23	2.98 ± 0.23
DSS	0.37 ± 0.13^###^	322.30 ± 26.57^###^	76.92 ± 9.50^###^	8.74 ± 1.51^###^
SASP	2.43 ± 0.80^*∗∗∗*^	357.20 ± 14.37^*∗∗*^	173.70 ± 24.49^*∗∗∗*^	3.38 ± 0.59^*∗∗∗*^
GQ	2.66 ± 0.67^*∗∗∗*^	360.70 ± 11.21^*∗∗*^	182.50 ± 43.52^*∗∗∗*^	3.54 ± 0.25^*∗∗∗*^

Note: ^#^*P* < 0.05, ^##^*P* < 0.01, and ^###^*P* < 0.0001 versus CON group. ^*∗*^*P* < 0.05, ^*∗∗*^*P* < 0.01, and ^*∗∗∗*^*P* < 0.0001 versus DSS group.

**Table 3:** Effect of GQ on the content of SOD, GSH, MDA, and ROS in the Caco-2 cells ($\bar{x}\pm s$, *n* = 3).

Groups	T-SOD/U·mL^−1^	GSH/*μ*mol·L^−1^	MDA/*μ*mol·L^−1^	ROS
CON	50.07 ± 4.01	141.08 ± 12.87	0.43 ± 0.17	10371.67 ± 632.62
MOD	30.83 ± 2.73^##^	43.37 ± 8.44^###^	1.95 ± 0.18^###^	38820.63 ± 1336.22^###^
BBR	41.40 ± 4.88^*∗*^	89.42 ± 4.08^*∗∗*^	1.03 ± 0.14^*∗∗*^	19678.67 ± 1000.07^*∗∗∗*^
L-GQ	30.53 ± 5.80	88.51 ± 5.99^*∗∗*^	1.83 ± 0.37	19645.33 ± 1041.39^*∗∗∗*^
M-GQ	38.68 ± 5.61^*∗*^	105.73 ± 5.99^*∗∗∗*^	1.04 ± 0.15^*∗∗*^	17008.07 ± 778.02^*∗∗∗*^
H-GQ	48.42 ± 6.54^*∗∗*^	137.81 ± 10.55^*∗∗∗*^	0.87 ± 0.17^*∗∗*^	9680.40 ± 263.37^*∗∗∗*^

Note: ^#^*P* < 0.05, ^##^*P* < 0.01, and ^###^*P* < 0.0001 versus CON group. ^*∗*^*P* < 0.05, ^*∗∗*^*P* < 0.01, and ^*∗∗∗*^*P* < 0.0001 versus MOD group.

**Table 4:** The effects of GQ on SOD (a), GSH (b), MDA (c), and ROS (d) were observed in Nrf2 silenced cells ($\bar{x}\pm s$, *n* = 3).

Groups	T-SOD/U·mL^−1^	GSH/*μ*mol·L^−1^	MDA/*μ*mol·L^−1^	ROS
CON	50.33 ± 4.62	141.66 ± 14.56	0.48 ± 0.19	10371.67 ± 623.62
SiNrf2	13.20 ± 2.56^###^	44.57 ± 7.36^###^	4.03 ± 0.11^###^	72151.63 ± 3380.41^###^
SiNrf2+GQ	24.70 ± 1.96^*∗∗*^	59.66 ± 8.54	2.43 ± 0.29^*∗∗*^	47596.57 ± 2570.21^*∗∗∗*^

Note: ^#^*P* < 0.05, ^##^*P* < 0.01, and ^###^*P* < 0.0001 versus CON group. ^*∗*^*P* < 0.05, ^*∗∗*^*P* < 0.01, and ^*∗∗∗*^*P* < 0.0001 versus SiNrf2 group.

**Table 5:** Effect of major components of GQ on content of SOD, GSH, MDA, and ROS ($\bar{x}\pm s$, *n* = 3).

Groups	T-SOD/U·mL^−1^	GSH/*μ*mol·L^−1^	MDA/*μ*mol·L^−1^	ROS
CON	49.99 ± 5.28	141.31 ± 13.69	0.43 ± 0.29	9959.70 ± 105.13
MOD	30.10 ± 2.52^##^	44.57 ± 7.36^###^	1.90 ± 0.29^###^	39305.17 ± 597.59^###^
GQ	41.18 ± 5.37^*∗*^	106.05 ± 6.79^*∗∗*^	0.97 ± 0.15^*∗∗*^	13791.20 ± 250.71^*∗∗∗*^
PUE	38.45 ± 4.67	94.01 ± 16.25^*∗∗*^	1.01 ± 0.15^*∗∗*^	19390.17 ± 158.45^*∗∗∗*^
BA	24.38 ± 3.07	52.85 ± 20.39	1.18 ± 0.10^*∗∗*^	23316.53 ± 522.65^*∗∗∗*^
BBR	41.03 ± 5.78^*∗*^	89.02 ± 4.92^*∗∗*^	1.09 ± 0.18^*∗∗*^	15060.37 ± 213.24^*∗∗∗*^
LQ	27.90 ± 3.21	59.99 ± 9.99	1.04 ± 0.09^*∗∗*^	28998.83 ± 831.95^*∗∗∗*^

Note: ^#^*P* < 0.05, ^##^*P* < 0.01, and ^###^*P* < 0.0001 versus CON group. ^*∗*^*P* < 0.05, ^*∗∗*^*P* < 0.01, and ^*∗∗∗*^*P* < 0.0001 versus MOD group.

## Data Availability

The datasets used and analyzed during the current study are available from the corresponding author on reasonable request.

## Supplementary Materials

Supplementary Materials Figure S1. Effect of GQ on the activity of Caco-2 cells. Table S1. RNA quality parameters of rats. Table S2. RNA quality parameters of Caco-2 cells after treatment by GQ. Table S3. RNA quality parameters of the Nrf2 gene silenced Caco-2 cells. Table S4. RNA quality parameters of Caco-2 cells after treatment by compounds of GQ. Table S5. Concentration of the analytes in samples of GQD and single drug sample (mg/g, *n* = 3).
