# Supplementary material for: Gegen Qinlian Decoction Relieves Ulcerative Colitis via Adjusting Dysregulated Nrf2/ARE Signaling
Source: Evid Based Complement Alternat Med. 2022 Apr 25;2022:2934552. doi: 10.1155/2022/2934552 (PMC9060978; doi:10.1155/2022/2934552)
Supplement: Supplementary Materials — Figure S1. Effect of GQ on the activity of Caco-2 cells. Table S1. RNA quality parameters of rats. Table S2. RNA quality parameters of Caco-2 cells after treatment by GQ. Table S3. RNA quality parameters of the Nrf2 gene silenced Caco-2 cells. Table S4. RNA quality parameters of Caco-2 cells after treatment by compounds of GQ. Table S5. Concentration of the analytes in samples of GQD and single drug sample (mg/g, n = 3). [file 2934552.f1.zip › 2934552.f1/Figure S1.docx]

Caco-2 cells were cultured with different concentrations of GQ for 24 hours. The concentration of GQ was 18.75 μg/ml, and the survival rate of Caco-2 cells was 81.63%. The concentration of GQ is 18.75 μg/ml and below did not affect the activity of Caco-2 cells.


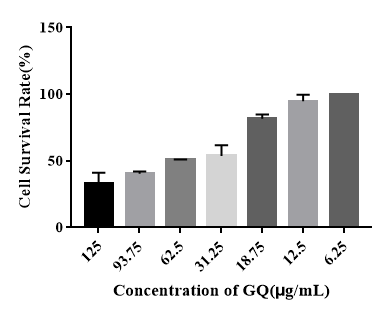


Figure S1. Effect of GQ on the activity of Caco-2 cells
