# Supplementary material for: Gegen Qinlian Decoction Relieves Ulcerative Colitis via Adjusting Dysregulated Nrf2/ARE Signaling
Source: Evid Based Complement Alternat Med. 2022 Apr 25;2022:2934552. doi: 10.1155/2022/2934552 (PMC9060978; doi:10.1155/2022/2934552)
Supplement: Supplementary Materials — Figure S1. Effect of GQ on the activity of Caco-2 cells. Table S1. RNA quality parameters of rats. Table S2. RNA quality parameters of Caco-2 cells after treatment by GQ. Table S3. RNA quality parameters of the Nrf2 gene silenced Caco-2 cells. Table S4. RNA quality parameters of Caco-2 cells after treatment by compounds of GQ. Table S5. Concentration of the analytes in samples of GQD and single drug sample (mg/g, n = 3). [file 2934552.f1.zip › 2934552.f1/Table S1.docx]

When OD_260_/OD_280_ (Ratio, R) in the RNA purity test is in the range of 1.8~2.1, we believe that protein contamination in RNA is acceptable. It can be seen from Table S1 that the purity of mRNA extracted from rats' colon tissue meets the requirements, and the next experiment can be carried out.

Table S1 RNA quality parameters of rats

| Sample | | A_260/280_ |
| --- | --- | --- |
| CON | 1 | 1.83 |
|  | 2 | 1.89 |
|  | 3 | 1.90 |
|  | 4 | 2.05 |
|  | 5 | 1.94 |
|  | 6 | 1.96 |
|  | 7 | 2.08 |
|  | 8 | 1.91 |
| DSS | 1 | 1.99 |
|  | 2 | 1.81 |
|  | 3 | 1.96 |
|  | 4 | 2.04 |
|  | 5 | 2.01 |
|  | 6 | 2.04 |
|  | 7 | 1.92 |
|  | 8 | 1.99 |
| DSS + SASP | 1 | 2.00 |
|  | 2 | 1.93 |
|  | 3 | 1.96 |
|  | 4 | 1.96 |
|  | 5 | 1.97 |
|  | 6 | 2.05 |
|  | 7 | 1.87 |
|  | 8 | 1.95 |
| DSS + GQ | 1 | 1.98 |
|  | 2 | 1.94 |
|  | 3 | 2.04 |
|  | 4 | 1.90 |
|  | 5 | 1.96 |
|  | 6 | 2.06 |
|  | 7 | 2.03 |
|  | 8 | 2.02 |
