# Supplementary material for: Gegen Qinlian Decoction Relieves Ulcerative Colitis via Adjusting Dysregulated Nrf2/ARE Signaling
Source: Evid Based Complement Alternat Med. 2022 Apr 25;2022:2934552. doi: 10.1155/2022/2934552 (PMC9060978; doi:10.1155/2022/2934552)
Supplement: Supplementary Materials — Figure S1. Effect of GQ on the activity of Caco-2 cells. Table S1. RNA quality parameters of rats. Table S2. RNA quality parameters of Caco-2 cells after treatment by GQ. Table S3. RNA quality parameters of the Nrf2 gene silenced Caco-2 cells. Table S4. RNA quality parameters of Caco-2 cells after treatment by compounds of GQ. Table S5. Concentration of the analytes in samples of GQD and single drug sample (mg/g, n = 3). [file 2934552.f1.zip › 2934552.f1/Table S3.docx]

When OD_260_/OD_280_ (Ratio, R) in the RNA purity test is in the range of 1.8~2.1, we believe that protein contamination in RNA is acceptable. It can be seen from table S3 that the purity of mRNA extracted from Nrf2 gene silenced Caco-2 cells meet the requirements, and the next experiment can be carried out.

Table S3 RNA quality parameters of Nrf2 gene silenced Caco-2 cells

| Sample | | A_260/280_ |
| --- | --- | --- |
| CON | 1 | 2.02 |
|  | 2 | 1.99 |
|  | 3 | 2.06 |
| SiNrf2 | 1 | 2.00 |
|  | 2 | 2.02 |
|  | 3 | 2.04 |
| SiNrf2 + GQ | 1 | 1.98 |
|  | 2 | 2.00 |
|  | 3 | 2.02 |
