# Supplementary material for: Gegen Qinlian Decoction Relieves Ulcerative Colitis via Adjusting Dysregulated Nrf2/ARE Signaling
Source: Evid Based Complement Alternat Med. 2022 Apr 25;2022:2934552. doi: 10.1155/2022/2934552 (PMC9060978; doi:10.1155/2022/2934552)
Supplement: Supplementary Materials — Figure S1. Effect of GQ on the activity of Caco-2 cells. Table S1. RNA quality parameters of rats. Table S2. RNA quality parameters of Caco-2 cells after treatment by GQ. Table S3. RNA quality parameters of the Nrf2 gene silenced Caco-2 cells. Table S4. RNA quality parameters of Caco-2 cells after treatment by compounds of GQ. Table S5. Concentration of the analytes in samples of GQD and single drug sample (mg/g, n = 3). [file 2934552.f1.zip › 2934552.f1/Table S5.docx]

Determination of components in GQ and determination of components of each herbal medicine in GQ by LC-MS / MS. The contents of flavonoids (baicalin, baicalein, wogonin, wogonin, wogonin, glycyrrhizin, glycyrrhizin), isoflavones (puerarin, daidzein, daidzein), alkaloids (berberine, jatrorrhizine, palmatine, berberine) and saponins (glycyrrhizic acid, glycyrrhetinic acid) were determined.

Table S5 Concentration of the analytes in samples of GQD and single drug sample (mg/g, n=3)

| Compound | GQ | Pueraria lobata (Willd.) Ohwi. | Scutellaria baicalensis Georgi. | Coptis chinensis Franch. | Glycyrrihiza uralensis Fisch. |
| --- | --- | --- | --- | --- | --- |
| Puerarin | 4.86 | 10.75 | － | － | － |
| Daidzin | 1.36 | 2.57 | － | － | － |
| Daidzein | 0.29 | 0.36 | － | － | － |
| Baicalin | 27.07 | － | 21.05 | － | － |
| Baicalein | 0.46 | － | 0.57 | － | － |
| Wogonoside | 10.59 | － | 14.15 | － | － |
| Wogonin | 0.18 | － | 0.22 | － | － |
| Liquiritin | 3.62 | － | －- | － | 4.31 |
| Liquiritigenin | 0.21 | － | － | － | 0.66 |
| Berberine | 6.76 | － | － | 9.65 | － |
| Jatrorrhizine | 2.34 | － | － | 3.68 | － |
| Palmatine | 3.34 | － | － | 6.10 | － |
| Coptisine | 6.47 | － | － | 8.36 | － |
| Glycyrrhizic acid | 1.71 | － | － | － | 3.22 |
